# Magnetic resonance imaging may simulate progressive multifocal leucoencephalopathy in a patient with chronic lymphocytic leukemia after fludarabine therapy

**DOI:** 10.4103/0972-2327.41880

**Published:** 2008

**Authors:** J. Kalita, N. S. Patel, U. K. Misra

**Affiliations:** Department of Neurology, Sanjay Gandhi Post Graduate Institute of Medical Sciences, Lucknow, India

**Keywords:** Progressive multifocal leucoencephalopathy, chronic lymphocytic leukemia, fludarabine

## Abstract

A 60-year-old male with chronic lymphatic leukemia (CLL) after 6 months of fludarabine therapy was admitted with status epilepticus and developed left hemiplegia. His magnetic resonance imaging revealed multiple T2 hyperintense lesions in the right frontal and left parieto-occipital lesion, simulating progressive multifocal leucoencephalopathy (PML). Cerebrospinal fluid Polymerase Chain Reaction (PCR) for JC virus was negative. We suggest the possible role of fludarabine in producing PML-like lesions in patients with Chronic Lymphocytic Leukemia (CLL).

## Introduction

Progressive multifocal leucoencephalopathy (PML) is a subacute demyelinating disease, resulting from the infection of oligodendrocytes by JC virus. It almost exclusively affects immunocompromised individuals and was first reported in a patient with chronic lymphocytic leukemia.[1] The widespread use of immunosuppression in the treatment of autoimmune diseases, organ transplantation and AIDS has resulted in the marked increase in its incidence and AIDS accounts for more than 85% cases of PML.[2] We recently managed a patient with Chronic Lymphocytic Leukemia (CLL) who manifested with status epilepticus, hemiplegia; his MRI simulated PML following the fludarabine therapy. We report this unusual manifestation and discuss the problem in diagnosis of PML.

## Case Report

A 60-year-old male who was suffering from CLL for the last 8 years developed anemia and thrombocytopenia 1.5 years back for which he received two courses of fludarabine. Following the therapy, his symptoms improved and he remained well for 6 months. Six months after the last course of fluderabine, he gradually became drowsy, and on the third day, he developed right partial seizures for 5–7 min, which was followed by right hemiplegia. Twelve hours later he had another partial seizure with secondary generalization that continued for 12 h. The patient did not have any past history of epilepsy, diabetes, hypertension or HIV.

On examination, he appeared to be a pale elderly male. Pulse was 80/min, BP was 120/80 mm of Hg and he had hepatosplenomegaly. The patient was comatose and responded to painful stimuli by limb extension. He had right-sided hemiplegia with hyperreflexia and right plantar response was extensor. The patient received IV fluids, intravenous ceftriaxone 2 g 12 hourly and IV phenytoin followed by sodium valproate for the control of status epilepticus. After 12 h, his seizures were controlled and consciousness started improving.

**Figure 1:**
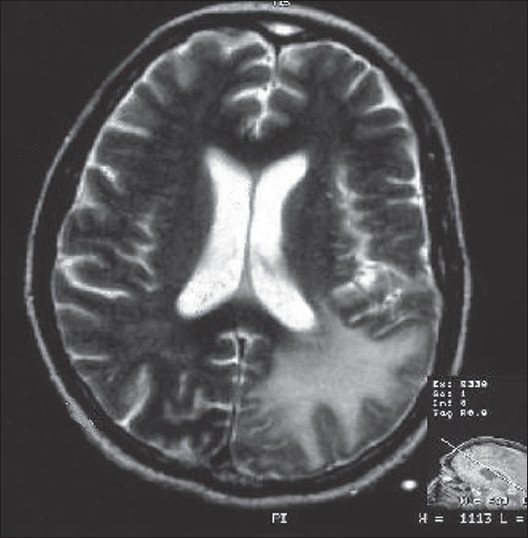
Cranial MRI, T2 sequence shows hyperintense subcortical lesion in left parietooccipital region in a patient with chronic lymphocytic leukemia, following fluderabine therapy

His hemoglobin was 9.5 g/dl, ESR was 13 mm for the first hour, total white cell count was 4000/mm^3^ with 90% polymorphs, 28% lymphocytes and 2% eosinophil and there were no immature cells. His blood sugar was 77 mg/dl, blood urea – 24 mg/dl, serum creatinine – 1.2 mg/dl, bilirubin – 1.2 mg/dl, SGOT – 471 U/L, SGPT – 41 U/L and the serum electrolyte, lipid profile, bleeding and coagulation parameters were normal. ANA, CRP, urinalysis and radiograph of chest were also normal. Electroencephalography showed diffuse theta slowing. His MRI in T1 sequence revealed subcortical left parieto-occipital and right frontal hypointensity without contrast enhancement or midline shift. The lesions were hyperintense on T2 sequence [Figure 1]. CSF was acellular with 45 mg% protein, 55 mg% sugar and was negative for bacteria and fungal culture. His HIV serology was negative and CSF PCR for JC virus was also negative. He was discharged after 35 days in a bed-ridden and confused state. At a three-month followup, there was no recurrence of seizures and he was able to talk, although bedridden.

## Discussion

Our patient with CLL after two courses of fluderabine therapy developed subacute encephalopathy characterized by altered sensorium, seizure and hemiplegia. His MRI was consistent with the diagnosis of PML and included multiple white matter lesions with scalloping of grey/white junction. The involvement of arcuate fibres, frontal and parietooccipital location of non-enhancing T1 hypointense and T2 hyperintense lesions have been reported to be highly suggestive of PML.[3] The occurrence of seizures particularly status epilepticus is a rare manifestation and has been reported in 10% patients with PML. The low sensitivity of PCR has been mainly attributed to the intracellular location of JC virus with only a few viruses in the CSF.[4] CSF PCR therefore has been regarded as additional but not sufficiently diagnostic. Stereotactic biopsy also has somewhat similar sensitivity and has been recommended when PCR is negative or if neuroimaging facilities are not available. Microscopy also helps in the diagnosis of another treatable lesion, but it is justified if a therapy is in sight.[5] We have, however, not done the biopsy because of the lack of advanced histological facilities.

In our patient, the PML-like lesions were noted following the fluderabine therapy. Similar lesions have been reported in untreated CLL patients and those treated with alkylating agents exclusively.[6,7] A patient with CLL developed a clinical picture simulating PML after 6 months of fluderabine therapy and died 8 months later.[8] In the light of such reports and our patient, the role of fluderabine in producing and promoting PML-like lesions in CLL needs further investigations.
